# *TP53* Mutation Is a Prognostic Factor in Lower Grade Glioma and May Influence Chemotherapy Efficacy

**DOI:** 10.3390/cancers13215362

**Published:** 2021-10-26

**Authors:** Humaira Noor, Nancy E. Briggs, Kerrie L. McDonald, Jeff Holst, Orazio Vittorio

**Affiliations:** 1 Cure Brain Cancer Biomarkers and Translational Research Group, Prince of Wales Clinical School, University of New South Wales, Sydney, NSW 2031, Australia; kezmcd275@gmail.com; 2 Adult Cancer Program, Lowy Cancer Research Centre, UNSW Sydney, Randwick, NSW 2031, Australia; j.holst@unsw.edu.au; 3 Stats Central, Mark Wainwright Analytical Centre, University of New South Wales, Sydney, NSW 2031, Australia; nancy.briggs@unsw.edu.au; 4 Translational Cancer Metabolism Laboratory, School of Medical Sciences, Prince of Wales Clinical School, UNSW Sydney, Sydney, NSW 2031, Australia; 5 School of Women’s & Children’s Health, UNSW Medicine, University of NSW, Randwick, NSW 2031, Australia; ovittorio@ccia.org.au; 6 Children’s Cancer Institute, Lowy Cancer Research Centre, UNSW Sydney, Randwick, NSW 2031, Australia

**Keywords:** *TP53* mutation, low-grade glioma, LGG, *MGMT* methylation, *YAP1*, chemosensitivity, temozolomide, prognostic factor, R273H, R273C

## Abstract

**Simple Summary:**

Molecular biomarkers are utilised for the development of targeted therapy and diagnostic or prognostic tools and in strategising therapeutic approaches as they may have an effect in drug response. In lower-grade glioma (LGG), there are currently no established biomarkers that are associated with chemosensitivity. Here, we identified *tumour protein 53 (TP53)* hotspot mutations in *TP53* codon 273 in 33% (17/51) of astrocytoma tissues and retrospectively found that these tumours were associated with significantly improved clinical outcomes when treated with chemotherapy. We used publicly available datasets to successfully confirm these findings. A potential mechanism of this chemosensitivity was explored in this study. *TP53* codon 273 mutations can, thus, potentially be an indicator of chemotherapeutic efficacy in astrocytoma, and it may be useful in making astrocytoma treatment decisions.

**Abstract:**

Background: Identification of prognostic biomarkers in cancers is a crucial step to improve overall survival (OS). Although mutations in *tumour protein 53* (*TP53*) is prevalent in astrocytoma, the prognostic effects of *TP53* mutation are unclear. Methods: In this retrospective study, we sequenced *TP53* exons 1 to 10 in a cohort of 102 lower-grade glioma (LGG) subtypes and determined the prognostic effects of *TP53* mutation in astrocytoma and oligodendroglioma. Publicly available datasets were analysed to confirm the findings. Results: In astrocytoma, mutations in *TP53* codon 273 were associated with a significantly increased OS compared to the *TP53* wild-type (HR (95% CI): 0.169 (0.036–0.766), *p* = 0.021). Public datasets confirmed these findings. *TP53* codon 273 mutant astrocytomas were significantly more chemosensitive than *TP53* wild-type astrocytomas (HR (95% CI): 0.344 (0.13–0.88), *p* = 0.0148). Post-chemotherapy, a significant correlation between *TP53* and *YAP1* mRNA was found (*p* = 0.01). In *O (6)-methylguanine methyltransferase (MGMT)* unmethylated chemotherapy-treated astrocytoma, both *TP53* codon 273 and *YAP1* mRNA were significant prognostic markers. In oligodendroglioma, *TP53* mutations were associated with significantly decreased OS. Conclusions: Based on these findings, we propose that certain *TP53* mutant astrocytomas are chemosensitive through the involvement of *YAP1*, and we outline a potential mechanism. Thus, *TP53* mutations may be key drivers of astrocytoma therapeutic efficacy and influence survival outcomes.

## 1. Introduction

Lower-grade gliomas (LGGs) are cancers of the central nervous system (CNS) that affect younger adults aged between 17 and 44 years, with the median overall survival (OS) ranging from 7 to 13 years [1]. The OS is relatively longer than other gliomas such as glioblastoma; however, since much younger adults are affected in LGG, and their lifespan is significantly cut short, LGG is a devastating disease. LGGs account for 19% of all gliomas [2] and consist of two main histologic subtypes—astrocytoma and oligodendroglioma. Efforts to identify an effective therapy for LGG persist; however, there have been no substantial improvements in the OS in the past 30 years [3]. Moreover, the current treatment strategy—surgery, chemotherapy and/or radiation [4]—can also negatively affect cognitive abilities and, hence, the quality of life of LGG patients [5].

Since the discovery of mutations in *isocitrate dehydrogenase enzyme isoform 1/2* (*IDH1/2*) and their prognostic relevance to LGG [6], *IDH1/2* inhibitors are currently being explored as a therapeutic option [7,8,9,10,11]. However, the discovery of additional prognostic markers may also assist in the development of effective drugs and to accurately analyse on-going (and retrospective) clinical trials to better understand responders and non-responders. Therefore, we set out to understand the prognostic effects and mechanisms of commonly occurring genetic alterations in LGG.

Molecular studies have confirmed the distinct genetic profiles of astrocytoma and oligodendroglioma, clearly indicating distinct pathways of gliomagenesis for these sub-types [12]. In recent years, since the discovery of *IDH1*/*2* mutation prevalence and its prognostic role in LGG, glioma classification has moved from histopathological features towards molecular characteristics [13]. This diagnostic shift has introduced further molecular sub-groups within astrocytoma and oligodendroglioma, which are useful in survival and drug response analyses of LGG in clinical trials. These molecular advancements also shed light on the spectrum of mechanisms utilised by these molecular sub-types and stress the need for targeted therapy. For instance, 1p/19q co-deletion is almost exclusively present in oligodendroglioma, while tumour protein 53 (*TP53*) mutation is prevalent in astrocytoma.

*TP53* is a tumour suppressor gene located in chromosome 17p13.1 that encodes the p53 protein [14]. The gene is involved in a number of crucial cellular processes such as cell cycle arrest, differentiation, apoptosis and DNA damage repair, thereby protecting the cell from tumourigenesis [15]. *TP53* mutations occur in 50% of all human cancers, rendering it the most common genetic alteration in cancer. Mutant p53 protein accumulates in the nucleus, with a longer half-life compared to WT p53, and the mutant protein interacts with oncoproteins, inducing tumourigenesis [16]. *TP53* mutation in LGG is an early event [17] occurring after *IDH1* mutation [18]. The *TP53* mutation rate in astrocytoma has been reported to be 50–75%, while in oligodendrogliomas, it is 10–34% [19,20]. *TP53* expression increases with increasing grades of oligodendroglioma [21]. However, no significant increase in p53 expression between increasing grades of astrocytoma has been reported [22,23]. The involvement of *TP53* in gliomagenesis may, thus, vary between the subtypes of LGG.

Despite the prevalence of *TP53* mutations in LGGs, their prognostic effects remain unclear. Prognostic effects of *TP53* mutations have been studied extensively; however, no concordance between the outcomes of individual studies was observed [19,24,25,26,27,28], possibly because the histological subtypes associated with different OS were combined as one cohort of either LGG or glioma, particularly in survival studies, which led to discordant results. Recent advancements in molecular classification and the availability of online cancer databases, which can be used to cross-check mutations to include only reported ‘pathogenic’ mutations, provide new opportunities to better determine the survival effects of *TP53* mutation in LGG.

In this paper, we investigate the prognostic effects of pathogenic *TP53* mutations in LGG and attempt to understand their mechanism of action. In order to determine the prognostic role of *TP53*, we have analysed a large cohort of LGG and designed our study to (i) perform separate survival analysis for astrocytoma and oligodendroglioma; (ii) include only ‘pathogenic’ *TP53* mutations in the survival analysis, cross-checked with COSMIC and NCBI databases; (iii) correlate *TP53* mutation with other molecular features; (iv) propose a mechanism of the prognostic effects based on *TP53* mutation status; and (v) understand the prognostic mechanism using available LGG datasets.

## 2. Materials and Methods

### 2.1. Tumour Sample Collection and Patient Population

A retrospective cohort of 102 LGG fresh frozen tumour specimens was obtained from the Steve and Lynette Waugh Brain Tumour Biobank, Lowy Cancer Research Centre, University of New South Wales. The patients in the cohort had undergone surgery between 2010 and 2018. The tumour inclusion criteria for the study were: (i) patients above the age of 18; (ii) diagnosed for grade II or grade III astrocytoma or oligodendroglioma; and (iii) patients who have signed an informed consent form for research use of the tumour samples. Approval from the University of New South Wales Ethics Committee was received for this study (Ethics Approval no. HC190501).

### 2.2. Clinical Data Collection

Clinical data of all patients were obtained from the Steve and Lynette Waugh Brain Tumour Biobank database. Clinical data included age at initial diagnosis, gender, dates of first and subsequent resections, types and dates of adjuvant therapies, extent of resection, OS duration and status of the patients, and progression-free survival (PFS) information. Tumour genetic information such as p53 expression, 1p/19q co-deletion status and *alpha thalassemia/mental retardation syndrome x-linked* (*ATRX)* status were obtained from available patient pathology reports. We also obtained proliferation index Ki67% information from pathology reports. The reports were reviewed systematically for the required information. Not all patients were tested for the same genetic variations. Clinical pathology reports of the majority of LGG patients include immunohistochemistry (IHC) staining for p53 expression (n = 62). Similarly, 1p/19q co-deletion is a commonly ordered LGG test in pathology and the data were available in the pathology reports for 49 LGG patients.

### 2.3. TP53 Sequencing

DNA from fresh frozen tissue samples was extracted using the DNEasy Blood and Tissue Kit (Qiagen), according to the manufacturer’s protocol. A polymerase chain reaction (PCR) was then performed for 10 exons of *TP53* using the forward and reverse primer sequences outlined in Supplementary File 1: Table S1. KapaTaq HiFi ReadyMix (High-fidelity) DNA polymerase was used for the reaction. PCR was performed under the following conditions: initial denaturation at 95 °C for 3 min, denaturation at 95 °C for 30 s, annealing at the temperature corresponding to the particular exon (Supplementary File 1: Table S1) for 30 s, followed by extension at 72 °C for 1 min. The PCR reaction was performed for 34 cycles. Agarose gel electrophoresis was used to visualise and confirm the success of the PCR reaction, and each PCR product was subsequently sent to the Australian Genome Research Facility (AGRF) for purification and Sanger sequencing. This method has 99.9% accuracy, and it is the gold standard for clinical research sequencing [29]. Sequencing was performed on both the forward and reverse strands of each exon in each sample. The sequencing files (.ab1 format) were analysed with the software Sequencher (version 5.4.6) to identify whether any mutations were present. The reference sequence used for *TP53* mutation analysis was NP_000537.3.

### 2.4. IDH1 Sequencing

PCR was performed to amplify the commonly mutated target region of *IDH1*, consisting of codon 132. The primer sequences were *Forward—*CGGTCTTCAGAGAAGCCATT and *Reverse—*GCAAAATCACATTATTGCCAAC. The PCR was performed under standard conditions: initial denaturation at 95 °C for 7 min, denaturation at 95 °C for 30 s, annealing at 54 °C for 30 s, followed by extension at 72 °C for 45 s. The PCR reaction was performed for 34 cycles. Agarose gel electrophoresis was used to visualise and confirm the success of the PCR reaction, and each PCR product was subsequently sent to the Australian Genome Research Facility (AGRF) for purification and Sanger sequencing. The sequencing files (.ab1) were analysed with the software Sequencher (version 5.4.6) to identify whether any mutations were present.

### 2.5. Determination of Pathogenic Mutations

The pathogenic status of the identified mutations was checked systematically. The mutation IDs for each of the *TP53* mutations found were located using COSMIC (https://cancer.sanger.ac.uk/cosmic, accessed on 18 May 2019) and NCBI (https://www.ncbi.nlm.nih.gov/gene, accessed on 18 May 2019) databases. The COSMIC and NCBI information pages for each of the mutations state a pathogenic score, which was used to validate the pathogenicity of each mutation. Only mutations with a reported pathogenic score of >0.99 were considered “pathogenic” and used in further analysis. One exception to this was a novel *TP53* mutation that was detected. The MutationTaster (http://www.mutationtaster.org/, accessed on 25 May 2019) platform was used to predict the pathogenicity of this novel mutation (occurring in a single oligodendroglioma patient).

### 2.6. Survival Analysis

OS was calculated from the date of histopathological diagnosis to the date of death, censored at the last follow-up. Time to first recurrence (considered as progression-free survival) was calculated from the date of first surgery to the date of the following surgery after radiological progression was confirmed. Patient vital status and date of last follow-up were last updated on 19 April 2019.

### 2.7. Survival Studies with Public Cancer Datasets

Survival analysis was performed on publicly available cancer datasets: The Cancer Genome Atlas (TCGA) [30], Memorial Sloan Kettering—IMPACT Clinical Sequencing Cohort (MSKCC) [31], and Chinese Glioma Genome Atlas (CGGA). cBioPortal (https://www.cbioportal.org/, accessed on 10 February 2021) was used to source mutation and clinical data for astrocytoma cases from TCGA and MSKCC datasets.

CGGA dataset was used due to their exclusive transcriptomics data availability for recurrent LGG tumours. Clinical and transcriptomic data (mRNAseq_693) [32,33] were downloaded directly from https://www.cgga.org.cn/ (accessed on 10 February 2021). All survival analysis was performed using IBM SPSS Statistics 26 software.

### 2.8. Determination of Prognostic Effects of Hippo Signalling Pathway Genes in TCGA Cancers and Their Correlation with TP53

Hippo Signalling pathway genes were selected for analysis from existing literature [34,35,36]. A log_10_ hazard ratio heatmap was used to visualise survival across TCGA cancers based on Hippo Signalling pathway gene expression, which was created using the survival function on GEPIA2 (http://gepia.cancer-pku.cn/, accessed on 21 February 2021). The correlation between Yes-associated Protein 1 (*YAP1*) and *TP53* mRNA across TCGA cancer types was analysed using the correlation function on GEPIA2 (http://gepia.cancer-pku.cn/, accessed on 21 February 2021). The distribution of Hippo Signalling pathway gene expression among astrocytoma *TP53* mutation statuses was visualised using heatmaps generated on cBioPortal (https://www.cbioportal.org/, accessed on 7 March 2021).

### 2.9. Differential Gene Expression, Gene Ontology and Pathway Analysis

Recurrent astrocytoma tumours treated with chemotherapy were selected from the CGGA dataset, and transcriptomics and clinical data were directly downloaded from the CGGA website. EdgeR was used for differentially expressed gene (DEG) analysis based on *YAP1* high and *YAP1* low groups. Genes with 1.5-fold upregulation or downregulation between *YAP1* high/*YAP1* low groups were considered differentially expressed. The cut off for *YAP1* high/*YAP1* low was set on the median expression mark, and a false discovery rate (FDR) <0.05 was considered significant. Enrichr [37] was used for gene ontology and KEGG pathway analysis. The analysis was conducted separately for upregulated and downregulated genes.

### 2.10. Protein–Protein Interaction Analysis

A search tool for the retrieval of interacting genes (STRING) (www.string-db.org, accessed on 9 March 2021) was used to determine protein–protein interaction (PPI). The significant DEG lists were submitted to the STRING website, and the PPIs were filtered to include only meaningful interactions that have been experimentally proven (total score > 0.4). This PPI network was saved and imported to Cytoscape [38], where module screening was performed with the molecular complex detection (MCODE) plugin [39] (scores > 3, nodes > 4). The top three PPI networks of modules were identified for each group.

### 2.11. Statistical Analysis

Survival probabilities (OS and PFS) were calculated using the Kaplan–Meier method, and a log-rank test was used to compare survival distributions (*p* < 0.05 considered significant). IBM SPSS Statistics (version 26) software was used to perform univariate survival analysis, and multivariate survival analysis with Cox proportional hazards model and a 2-sided *p*-value of less than 0.05 was considered statistically significant. Multivariate analysis was performed to adjust for prognostic effects of *IDH1* mutation, age, sex and grade (also 1p/19q co-deletion for the TCGA oligodendroglioma dataset). GraphPad Prism 8.0 software was used to plot mRNA expression correlations, distribution of age and Ki67 and p53 expression across *TP53* mutation status. Student’s *t*-test was used to determine significance.

## 3. Results

### 3.1. Tumour Cohort Characteristics

This study investigated a cohort of 102 LGG, including 51 astrocytomas (32 grade II and 19 grade III) and 51 oligodendrogliomas (26 grades II and 25 grade III). The median age of the cohort of patients was 36 ± 10 (*Std*), which is within the typical age range for LGG patients [39]. The gender ratio in our study population was 1.1 (53 females/49 males). The overall median survival of the astrocytoma cohort was 123 months; for the oligodendroglioma cohort, it was 159 months (Supplementary File 2: Figure S1). *IDH1* mutation was present in 83% (85/102) of the tumours in the LGG cohort. Our results confirmed previous reports that the *IDH1* R132H mutation is the most common form of *IDH1* mutation in LGG as all of the cases were R132H mutants except two, which were both R132C mutants [6,40] (Table 1). LGG tumours most commonly occurred in the fronto-temporal region of the brain (64% cases), which is also in accordance with known literature [41]. All patients underwent gross total resection. Following surgery, the patients received adjuvant therapies including chemo-radiation therapy (27.5%), radiation therapy (10.8%) or chemotherapy (14.7%); however, 26.5% of patients did not receive adjuvant therapy after surgical resection (Table 1).

### 3.2. Population Survival Characteristics

The median OS of the cohort was 13.3 years (95% CI: 9.5–16.8 years) based on 38 deaths. The Kaplan–Meier curves in Supplementary File 2: Figure S1 shows the survival of patients based on their *IDH1* mutation status, histological type, and *TP53* mutation status (to be discussed in the following section). As expected from a typical LGG cohort, our cohort showed that IDH-mutant cases corresponded to a significantly increased OS (*IDH1* WT vs. MUT median OS: 67.5 mo. vs. 168 mo.; HR (95% CI): 3.65 (1.33–10.00), *p* < 0.0001) (Supplementary File 2: Figure S1A). Additionally, expectedly, oligodendroglioma patients showed a significantly increased OS compared to astrocytoma patients (oligodendroglioma vs. astrocytoma median OS: 159 mo. vs. 123 mo.; HR (95% CI): 0.52 (0.27–0.98), *p* = 0.045) (Supplementary File 2: Figure S1B).

### 3.3. TP53 Mutation Types and Prevalence in LGG

*TP53* exons 1–10 were sequenced in the cohort of LGG, and a mutational hotspot region of exons 4–8 was noted. However, the mutational hotspot region for ‘pathogenic’ *TP53* mutation (as determined from COSMIC and NCBI pathogenic scores >0.99) was between exons 5 to 8. Table 2 reports 27 different mutations identified, which include missense, nonsense, silent and frameshift deletion mutations. A total of 21 out of the 27 mutations were reportedly ‘pathogenic’. There was no case where two pathogenic mutations were present in one tumour specimen. Considering only pathogenic *TP53* mutations, 60% (31/51) of astrocytomas and 18% (9/50) of oligodendrogliomas were *TP53* mutants (*TP53* mutation status could not be determined in one oligodendroglioma tumour) (Table 3). The non-pathogenic rs1042522 polymorphism was present in 24 LGGs (Table 2).

**Figure 1:**
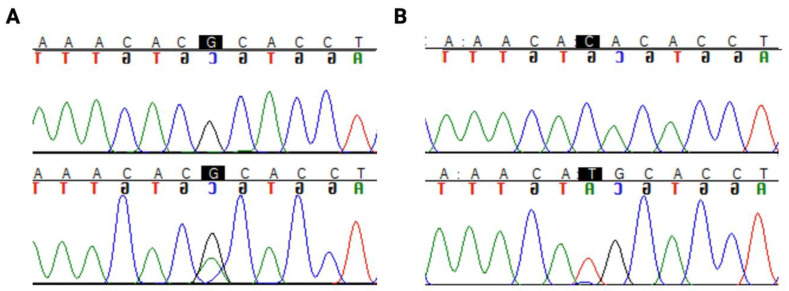
*TP53* hotspot mutations in codon 273: (**A**) R273C (c.817C>T) (**B**) R273H (c.818G>A). The nucleotide of interest is highlighted in black. The top row of the sequence is the reference sequence, and the bottom row is the mutant sequence.

Interestingly, a hotspot mutation region in codon 273 (Figure 1) was identified in 33% (17/51) of astrocytomas. Only 1 out of 50 oligodendrogliomas harboured a mutation in this codon. There were two pathogenic mutations in this hotspot region, R273C (c.817C>T) and R273H (c.818G>A), as shown in Figure 1.

### 3.4. Correlation of TP53 Mutation with Other Clinicopathological Features

We next compared the clinicopathological features of *TP53* mutation status (Table 3). *TP53* mutation status significantly varied between histological subtypes of LGG (*p* < 0.001). *TP53* mutant patients were significantly younger than *TP53* wild-type (WT) patients (34 vs. 38 years) (*p* = 0.046). *IDH1* mutation status, p53 expression status (*p* > 10% is considered ‘high’) and 1p/19q co-deletion status were significantly different among *TP53* mutant and *TP53* WT groups (*p* = 0.009, *p* < 0.001 and *p* < 0.001, respectively). In *TP53* mutant cases, there was higher expression of p53 protein, and 37/39 (94.8%) *TP53* mutant cases were also *IDH1* mutants. Previous reports showed that 1p/19q co-deletion was mutually exclusive to *TP53* mutation [40]. However, 1p/19q co-deletion status was unknown for a number of patients in our cohort, and, hence, the mutual exclusivity cannot be verified in our study. Treatment received was significantly different among *TP53* mutant and WT patients (*p* = 0.016); however, this cannot be concluded due to the high frequency of patients with unknown treatment types in these groups. There was no significant difference between the gender of *TP53* mutant patients and WT patients (Table 3).

### 3.5. Prognostic Effects of TP53 Mutations in LGG

#### 3.5.1. Prognostic Effects of *TP53* Mutations in Astrocytoma

When the cohorts of astrocytoma and oligodendroglioma were combined as ‘LGG’, expectedly, because of the different OS of each of the subtypes, no significant prognostic effect of *TP53* mutation was observed (*p* = 0.917) (Supplementary File 2: Figure S1C).

**Figure 2:**
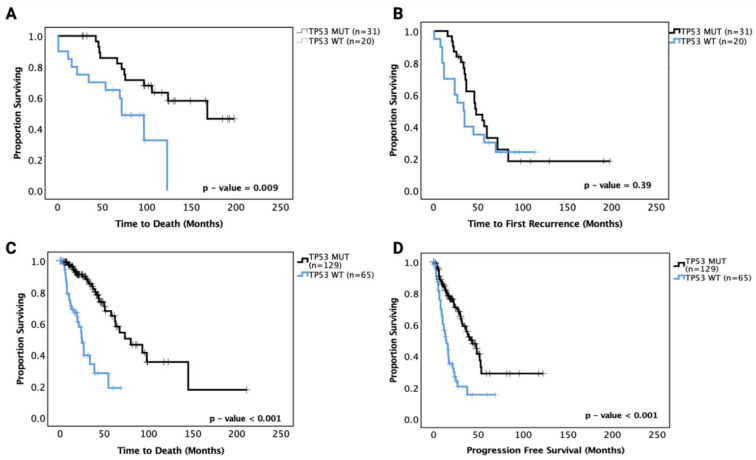
Kaplan–Meier survival curves for grades II and III astrocytoma in our cohort and the TCGA astrocytoma dataset based on *TP53* mutation status. (**A**) Overall survival and (**B**) progression-free survival curves for our astrocytoma cohort. (**C**) Overall survival and (**D**) progression-free survival curves for the TCGA astrocytoma cohort. WT: wild-type, MUT: *TP53* mutant. Log-rank test was used for analysis and a *p*-value < 0.05 for statistical significance.

We then performed survival analysis on the astrocytoma and oligodendroglioma cohorts separately. In astrocytoma, ‘pathogenic’ *TP53* mutant patients showed a significantly prolonged OS (median OS of *TP53* MT 165 mo. vs. *TP53* WT 70 mo., *p* = 0.009) compared to *TP53* WT patients in univariate analysis (Figure 2A). In multivariate analysis adjusting for *IDH1* mutation status, age, gender and grade, however, the effect was no longer significant (*p* = 0.091). PFS analysis showed no significant prognostic effect of *TP53* mutation (*p* = 0.39) (Figure 2B). These data suggest that there may be a sub-group within the *TP53* mutations responsible for the significantly improved survival in univariate analysis. The hazard ratios and 95% confidence intervals are detailed in Table 4 for the univariate and multivariate analyses.

The TCGA LGG dataset was used to confirm these prognostic effects. Grades II and III astrocytoma cases from the dataset were analysed for OS and PFS based on *TP53* mutation status, and the results mirrored the OS findings from our cohort with *TP53* mutant cases, showing significantly increased OS (median survival *TP53* WT 30 months vs. *TP53* MT 75 months, *p* < 0.001). Further, the TCGA LGG astrocytoma dataset showed significantly improved PFS for *TP53* mutant cases (*TP53* WT 18 months vs. *TP53* MT 42 months, *p* < 0.001) (Figure 2C,D). It should be noted that the *TP53* mutations in the TCGA dataset may include unknown numbers of non-pathogenic mutations that may affect the overall effect sizes. It should also be noted that the OS and PFS of the TCGA astrocytoma dataset were shorter than the OS and PFS of the current cohort, which may be because the TCGA dataset includes specimens with older dates of diagnosis and treatment strategies (1992 to 2013) than the current cohort (2010 to 2018).

**Figure 3:**
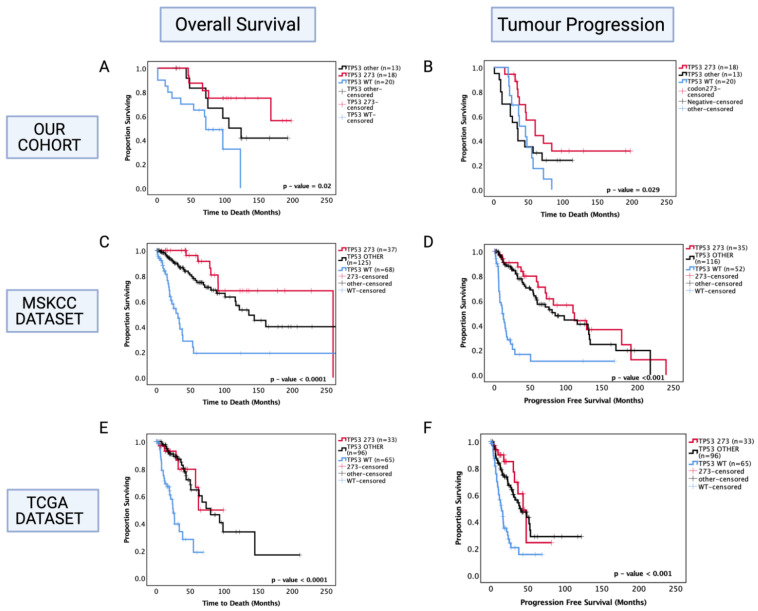
Kaplan–Meier survival curves for astrocytoma based on *TP53* codon 273 mutation, other *TP53* mutations, and *TP53* wild-type status. (**A**) Overall survival and (**B**) progression-free survival stratified by *TP53* wild-type, *TP53* codon 273 mutant, and other *TP53* mutants in our dataset. (**C**) Overall survival and (**D**) progression-free survival curves for the MSKCC dataset. (**E**) Overall survival and (**F**) progression-free survival curves for the TCGA astrocytoma cohort stratified by *TP53* wild-type, *TP53* codon 273 mutant, and other *TP53* mutants in the TCGA dataset. *TP53* 273: *TP53* codon 273 mutant, *TP53* OTHER: all pathogenic *TP53* mutations except in codon 273, *TP53* WT: wild-type *TP53* status. Log-rank test was used for analysis and a *p*-value < 0.05 for statistical significance.

Following the findings from combined *TP53* pathogenic mutations, we next investigated the prognostic role of specific commonly occurring hotspot mutations in codon 273 (shown previously in Figure 1) to determine whether this sub-group was more prognostically relevant than the diverse combination of other *TP53* mutations. Thus, the astrocytoma cohort was further divided into codon 273 mutants, *TP53* other mutants (that include patients with all other pathogenic *TP53* mutations) and *TP53* WT categories. In univariate analysis, only codon 273 mutant cases corresponded to a significantly increased OS (50% survival mark not reached for codon 273 vs. *TP53* WT 65 months, *p* = 0.011) and PFS (codon 273 65 months vs. *TP53* WT 45 months, *p* = 0.029) (shown in Figure 3A,B) compared to *TP53* WT, while ‘other’ combined pathogenic *TP53* mutations were not a prognostic factor (*p* = 0.241 and *p* = 0.951). No significant difference was observed between other *TP53* mutant and codon 273 mutant groups, suggesting that possibly other infrequent mutations that are prognostic factors may be present within the combined ‘other’ group, which remains to be characterised. In the multivariate analysis of PFS, *TP53* codon 273 mutants were no longer a significant prognostic factor; however, multivariate analysis confirmed that *TP53* mutation in codon 273 is an independent prognostic factor for OS in astrocytoma (HR (95% CI): 0.169 (0.036–0.766), *p* = 0.021) (Table 4).

In the MSKCC astrocytoma dataset, both ‘other’ *TP53* and codon 273 mutant cases showed improved OS and PFS compared to WT, with the codon 273 mutant group showing the most improved OS and PFS among the three groups (Figure 3C,D) (OS: WT = 30 mo., other = 140 mo., codon 273 = 260 mo., *p* < 0.0001) (PFS: WT = 10 mo., other = 80 mo., codon 273 = 110 mo., *p* < 0.001).

In the TCGA cohort, both ‘other’ *TP53* mutants and codon 273 mutants showed a significantly improved OS (WT: 25 mo., other: 75 mo., 273: not reached, *p* < 0.0001) and PFS (WT: 18 mo., other: 48 mo., 273: 48 mo., *p* < 0.001) compared to WT (Figure 3E,F).

Again, it should be noted here that in the TCGA dataset, all *TP53* mutations were considered for analysis as it was not possible to determine ‘pathogenic’-only mutations. Hence, non-pathogenic *TP53* mutations were inherently included in the analysis, which may have augmented the OS distribution of the ‘other’ *TP53* mutant category. Table 5 summarises the hazard ratios and 95% confidence intervals for MSKCC and TCGA survival analysis.

#### 3.5.2. Prognostic Effects of *TP53* Mutations in Oligodendroglioma

**Figure 4:**
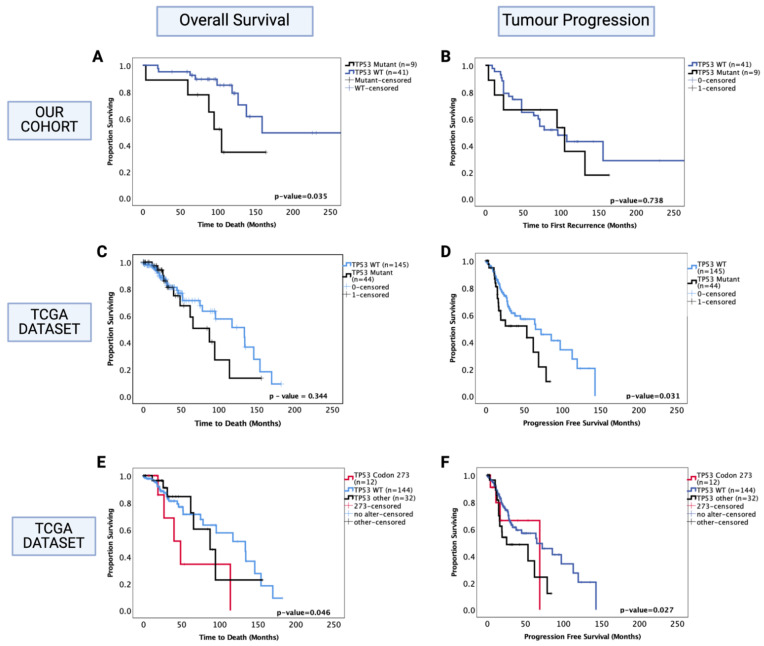
Kaplan–Meier survival curves for oligodendroglioma based on *TP53* mutation status. (**A**) Overall survival and (**B**) progression-free survival stratified by *TP53* wild-type and *TP53* mutant cases in our dataset. (**C**) Overall survival and (**D**) progression-free survival curves for the TCGA oligodendroglioma dataset, stratified by *TP53* wild-type and *TP53* mutant cases. (**E**) Overall survival and (**F**) progression-free survival curves for the TCGA oligodendroglioma dataset, stratified by *TP53* wild-type, *TP53* codon 273 mutant, and other *TP53* mutant cases. *TP53* 273: *TP53* codon 273 mutant, *TP53* OTHER: all pathogenic *TP53* mutations except in codon 273, *TP53* WT: wild-type *TP53* status. Log-rank test was used for analysis and a *p*-value < 0.05 for statistical significance.

Separate survival analysis was conducted on the oligodendroglioma cohort to determine the role of *TP53* mutation as a prognostic factor in this histological sub-type of LGG. *TP53* mutation status was successfully determined in 50 out of 51 oligodendroglioma tumours, and 18% (9/50) of the tumours were *TP53* mutants. Only one out of nine *TP53* mutations were in codon 273 in our cohort. We found that the presence of *TP53* mutation was associated with significantly decreased OS of oligodendroglioma patients (*TP53* mutant vs. WT median survival: 100 mo. vs. 160 mo., HR (95% CI): 3.09 (1.023–9.32), *p* = 0.035) (Figure 4A). In multivariate analysis, *TP53* mutation remained a prognostic factor (HR (95% CI): 6.52 (1.71–24.89), *p* = 0.006); however, it should be noted that 1p/19q co-deletion was not accounted for in the multivariate analysis in our cohort since this information was not available for all patients. There was a total of 30 cases with known 1p/19q co-deletion status (9 had no co-deletion and 21 were co-deleted), and 3 out of 9 non-co-deleted tumours had *TP53* mutation whereas 2 out of 21 co-deleted tumours had *TP53* mutation. No significant prognostic effect was observed in the PFS (*p* = 0.742) (Figure 4B).

The oligodendroglioma dataset from the TCGA was used to confirm these findings. In this dataset, 23% (44/189) oligodendroglioma tumours were *TP53* mutants, of which 12 were mutations in the *TP53* codon 273. The 1p/19q co-deletion status was known for all samples; hence, the multivariate analysis included adjustments for this molecular marker. We found no significant prognostic effect of *TP53* mutation for OS (*p* = 0.344) (Figure 4C); however, the presence of *TP53* mutation was associated with a significantly decreased PFS (Figure 4D) (*TP53* mutant vs. WT, median PFS: 55 mo. vs. 75 mo., HR (95% CI): 1.78 (1.05–3.03), *p* = 0.031). In multivariate analysis (1p/19q co-deletion accounted for), this effect was no longer significant (HR (95% CI): 1.06 (0.49–2.31), *p* = 0.837).

We next analysed the codon 273 mutants separately from other *TP53* mutations and found that only codon 273 mutants were associated with significantly decreased OS (codon 273 vs. WT median OS: 48.7 mo. vs. 133.7 mo., HR (95% CI): 2.63 (1.019–6.95), *p* = 0.046) (Figure 4E), while only other *TP53* mutants were associated with significantly decreased PFS compared to *TP53* WT (*TP53* other vs. WT median PFS: 23 mo. vs. 65 mo., HR (95% CI): 1.89 (1.07–3.37), *p* = 0.027) (Figure 4F). In multivariate analysis, other *TP53* mutations only showed a trend of decreased PFS (*p* = 0.056); however, codon 273 mutation remained a significant prognostic factor for oligodendroglioma OS in multivariate analysis (HR (95% CI): 9.05 (2.39–34.31), *p* = 0.001). Thus, *TP53* codon 273 mutation is a key prognostic event in both astrocytoma and oligodendroglioma, having opposite effects on the survival of each sub-type. In the TCGA oligodendroglioma dataset, 9 out of 12 codon 273 mutations were present in the 1p/19q non-co-deleted sub-group and 3 out of 12 in the 1p/19q co-deleted sub-group; moreover, 57% (25/44) of *TP53* mutant oligodendroglioma were 1p/19q non-co-deleted (total non-co-deleted samples in this dataset were n = 40). Hence, the observed decrease in OS in oligodendroglioma could be due to 1p/19q co-deletion or *TP53* mutation. Due to this reason, we could not confirm whether *TP53* mutation was an independent prognostic factor. Thus, we did not proceed with further investigation of the possible mechanism involved in oligodendroglioma. Nevertheless, the TCGA dataset findings show that although both codon 273 and other *TP53* mutations were associated with 1p/19q co-deletion, it was only the codon 273 mutation in particular that corresponded to a decreased OS, establishing its importance as a prognostic factor and potential therapeutic target. Since only one tumour was a codon 273 mutant in our cohort and 1p/19q co-deletion status was missing for a number of cases, we could not confirm these findings in our cohort.

### 3.6. TP53 Mutation Is Associated with Elevated p53 Expression

**Figure 5:**
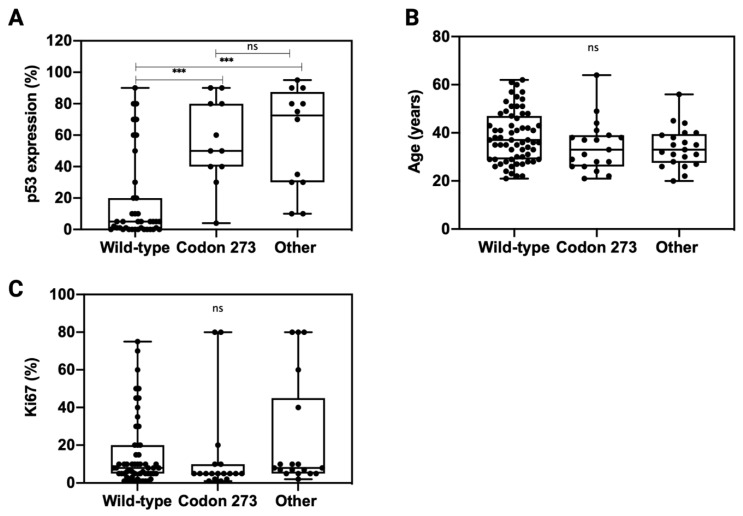
Distribution of p53 expression (**A**), patient age (**B**) and Ki67 (**C**) across *TP53* mutation types: codon 273 mutant, wild-type and other *TP53* mutations. Unpaired *t*-test was used for statistical analysis. *** *p* < 0.001, ns: not significant.

In order to understand whether *TP53* codon 273 mutations affect the expression of p53, we analysed p53 expression across *TP53* mutation categories (Figure 5A). Compared to *TP53* WT, both other *TP53* mutations and *TP53* codon 273 mutations correlated with a significantly higher p53 expression (*p* < 0.001). There was no significant difference in the p53 expression levels between codon 273 mutants and other *TP53* mutant tumours (*p* = 0.94). The patient age and tumour proliferation index, Ki67% (data extracted from the pathology report), was similar across all *TP53* mutant and WT categories (Figure 5B,C).

As no significant difference was observed between *TP53* expression levels in the other *TP53* mutant and codon 273 mutant groups, the mechanism through which codon 273 improves survival compared to the other mutant groups likely involves factors more than just the expression modulation of mutant p53 proteins. As the tumour proliferation rate was similar between codon 273 mutant, other *TP53* mutant and *TP53* WT groups, it suggests that the improved survival of codon 273 mutant patients might not be due to decreased tumour proliferation activity. Codon 273 mutant patients were not significantly younger than the other groups; hence, the improved survival was not related to patient age.

### 3.7. Enhanced Chemosensitivity of Codon 273 Mutant Astrocytoma May Lead to Improved Overall and Progression-Free Survival

**Figure 6:**
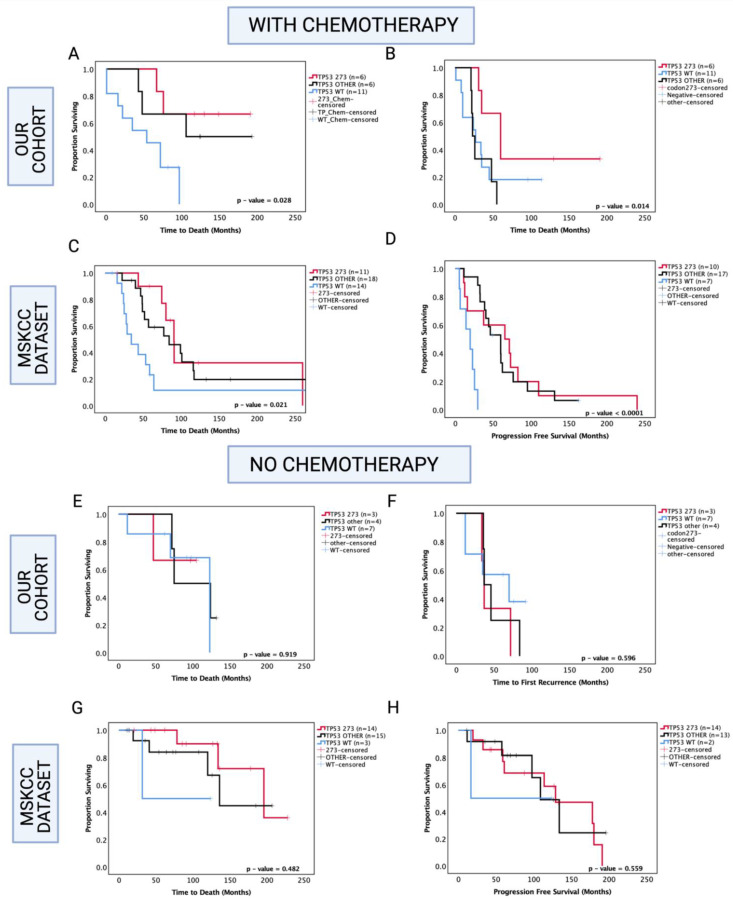
Kaplan–Meier survival curves comparing effects of *TP53* mutation status in the prognosis of chemotherapy-treated and no-chemotherapy groups. OS and PFS of chemotherapy-treated groups in our cohort (**A**,**B**) and the MSKCC cohort (**C**,**D**). OS and PFS of no-chemotherapy groups in our cohort (**E**,**F**), and the MSKCC cohort (**G**,**H**). *TP53* 273: *TP53* codon 273 mutant, *TP53* OTHER: all pathogenic *TP53* mutations except in codon 273, *TP53* WT: wild-type *TP53* status. Log-rank test was used for analysis and a *p*-value < 0.05 for statistical significance.

Since chemotherapy is commonly administered in astrocytoma, one possibility is that codon 273 mutant patients are more sensitive to therapy compared with other *TP53* mutant patients and *TP53* WT. Hence, in our analysis, we next set out to determine the role of *TP53* mutations in inducing chemosensitivity (Figure 6). In our cohort of known chemotherapy status, other *TP53* mutations were associated with a trend toward increased OS within the chemotherapy-treated group (other *TP53* vs. WT median survival: 149.5 mo. vs. 54 mo., HR (95% CI): 0.329 (0.11–1.02), *p* = 0.054), and *TP53* codon 273 mutations were associated with significantly increased OS compared to *TP53* WT in the chemotherapy-treated group (codon 273 vs. WT median survival: not reached vs. 54 mo., HR (95% CI): 0.225 (0.07–0.73), *p* = 0.028 (Figure 6A). It should be noted that in the chemotherapy group, 8/23 patients had received only chemotherapy and 15/23 patients were also radiotherapy-treated, and within the no-chemotherapy group, 9/14 patients underwent gross total resection only and 5/14 patients were radiotherapy-treated. Thus, the role of radiotherapy remains unclear in our analysis. In the PFS analysis within the chemotherapy-treated group, other *TP53* mutations were not significantly associated with progression (*p* = 0.788); however, codon 273 mutation showed a trend toward increased PFS (codon 273 vs. WT median survival: 60 mo. vs. 24.5 mo., HR (95% CI): 0.438 (0.14–1.29), *p* = 0.071) compared to WT. Codon 273 mutation was associated with significantly increased PFS compared to other *TP53* mutations (codon 273 vs. other *TP53* median survival: 60 mo. vs. 24.5 mo., HR (95% CI): 0.235 (0.06–0.99), *p* = 0.014) (Figure 6B). This indicates possible chemosensitivity of *TP53* codon 273 mutants and possibly some of the ‘other’ *TP53* mutant tumours.

In the MSKCC cohort of chemotherapy-treated astrocytoma, both codon 273 and other *TP53* mutations were associated with significantly increased OS (other vs. WT median survival: 84.2 mo. vs. 34.4 mo., HR (95% CI): 0.459 (0.19–0.93), *p* = 0.031; codon 273 vs. WT median survival: 90.6 vs. 34.4 mo., HR (95% CI): 0.344 (0.13–0.88), *p* = 0.0148) and significantly increased PFS (other vs. WT median PFS: 53.5 mo. vs. 19.6 mo., HR (95% CI): 0.177 (0.04–0.96), *p* < 0.0001; codon 273 vs. WT median PFS: 68.4 mo. vs. 19.6 mo., HR (95% CI): 0.332 (0.09–0.63), *p* = 0.009) (Figure 6C,D).

Interestingly, within the group of patients who did not receive chemotherapy, in our cohort and the MSKCC cohort, there were no significant associations between *TP53* mutations and OS/PFS (Figure 6E–H).

Hence, our findings indicate that codon 273 mutant tumours are possibly more chemosensitive than *TP53* WT and ‘other’ *TP53* mutant groups, although, within the ‘other’ mutant group, there may be a number of less frequently occurring mutations that give the same prognostic effect as codon 273, ultimately leading to the *TP53* other group trending toward an improved clinical outcome. It is, thus, important to understand the effects of individual *TP53* mutation types/codons.

We next set out to examine whether the chemosensitivity mechanism may involve the binding of transactivated isoforms (TA) of p73 (other members of the p53 family) with *yes-associated protein 1*, *YAP1*. *YAP1* (a core component of the Hippo pathway) is a transcriptional co-activator of TAp73, and p300-mediated acetylation of TAp73 leads to its increased binding with *YAP1*, resulting in TA-p73/*YAP1* complexes to promote apoptosis through several target apoptotic genes [41]. Furthermore, *YAP1* stabilises p73 in response to chemotherapy and DNA damage, enhancing its apoptotic function [42] and affinity to p73 [43]. The YAP-p73 complex has been shown to activate treatment-induced apoptosis in colorectal cancer, breast cancer and other malignancies [44,45,46]. Hence, we analysed the correlation of *YAP1* with *TP53* mRNA expression and its prognostic involvement in astrocytoma.

### 3.8. YAP1 mRNA Expression Is Associated with TP53, and Hippo Signalling Pathway Genes Are Prognostic Factors in LGG

**Figure 7:**
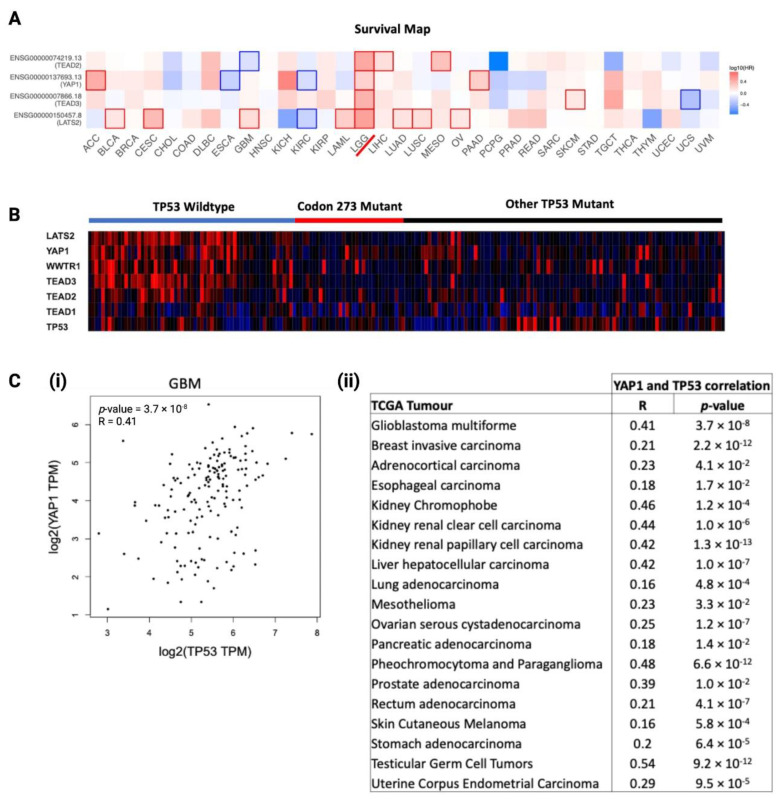
Association between *TP53* mutation/mRNA expression with *YAP1* mRNA expression, and the prognostic significance of *YAP1* in cancers. (**A**) Log_10_ hazard ratio (log_10_HR) of *YAP1* mRNA expression (median cut-off) in TCGA cancers. LGG is marked with a red line. Statistically significant log_10_ hazard ratios (*p* < 0.05) are outlined with solid borders. (**B**) Hippo Signalling pathway and *TP53* gene expression heatmap distribution among *TP53* mutation statuses in TCGA astrocytoma as a gradient of high expression (red), neutral expression (black) and low expression (blue). (**C**) (**i**) Correlation between *YAP1* and *TP53* mRNA expressions in GBM. (**ii**) Correlation between *YAP1* and *TP53* mRNA expressions in selected TCGA cancers.

We analysed the prognostic significance of core Hippo Signalling pathway genes, determined from existing literature [34,35,36], among TCGA cancers, visualised as a heatmap of log_10_(HR) in Figure 7A. LGG was the only cancer where key Hippo Signalling pathway genes were significant prognostic markers, which further substantiated the rationale for investigating the interaction between *TP53* and *YAP1* post-chemotherapy. To investigate the distribution of Hippo Signalling pathway gene mRNA expression among *TP53* mutants and *TP53* WT astrocytoma, we generated a heatmap to visualise TCGA astrocytoma samples in Figure 7B. Clearly, the *TP53* WT astrocytoma group showed the highest expression of Hippo Signalling pathway genes. Codon 273 mutant and other *TP53* mutant groups showed no clear difference in the gene expression profile, indicating that some of the other *TP53* mutations may have similar interactions with the Hippo pathway. In order to understand the relationship between *YAP1* and *TP53*, we analysed the correlation between the mRNA of these two genes within TCGA cancers. We found 19 cancer types showing a significant positive correlation between *YAP1* and *TP53* mRNA expression in primary tumours (summarised in Figure 7C(i)). The correlation plot for GBM is presented in Figure 7C(ii). LGG was not in the list of cancers showing a positive correlation, which was not unexpected, considering the significant association between these genes was observed in recurrent LGG tumours only.

### 3.9. YAP1 mRNA Expression Levels Are Prognostic in Only Chemotherapy-Treated Recurrent Astrocytoma

To test the hypothesis of *YAP1* involvement in *TP53* mutation-mediated chemosensitivity, we investigated this mechanism in silico, using the CGGA dataset that includes clinical and transcriptomics data on recurrent gliomas. This dataset was used because recurrent LGGs were available in this dataset, unlike TCGA and MSKCC datasets, where only primary LGG data were available. Our investigation required recurrent tumours for the analysis of post-treatment effects. Investigating recurrent astrocytoma samples that were specifically treated with chemotherapy was necessary to understand the role of chemotherapy on *YAP1*.

**Figure 8:**
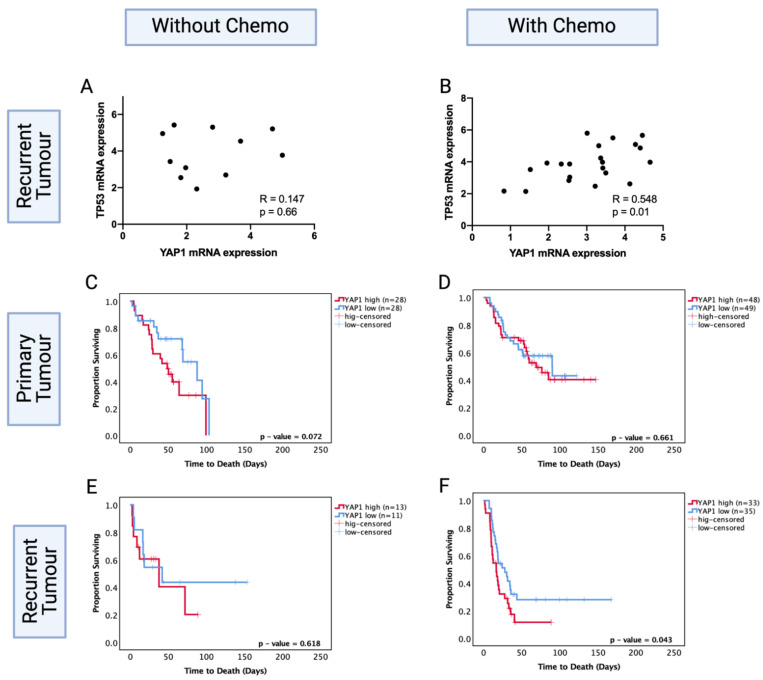
*TP53* mRNA levels in *YAP1* high and low groups in the CGGA dataset in patients (**A**) without chemotherapy and (**B**) with chemotherapy. Kaplan–Meier survival curves comparing effects of *YAP1* mRNA levels in the prognosis of (**C**) no-chemotherapy and (**D**) chemotherapy-treated groups in primary tumours. Kaplan–Meier survival curves comparing effects of *YAP1* mRNA levels in the prognosis of (**E**) no-chemotherapy and (**F**) chemotherapy-treated groups in recurrent tumours. Log-rank test was used for analysis and a *p*-value < 0.05 for statistical significance.

While specific *TP53* mutation data were not available for the CGGA dataset, thereby limiting our capacity to directly analyse the relationship with *TP53* codon 273 mutation, we found that *TP53* mRNA expression positively correlated with *YAP1* levels in the chemotherapy group. The high *YAP1* level group showed significantly higher levels of *TP53* mRNA expression (R = 0.548, *p* < 0.05). However, the recurrent astrocytoma group that did not receive chemotherapy showed no significant correlation between *TP53* and *YAP1* mRNA expression levels (R = 0.147, *p* = 0.66) (Figure 8A,B). This suggests a role for chemotherapy in modulating the interaction of *TP53* and *YAP1*.

We next investigated the prognostic role of *YAP1* levels in primary and recurrent astrocytoma with and without chemotherapy. Within the primary tumours, *YAP1* mRNA expression levels were not a significant prognostic factor with (*p* = 0.66) or without (*p* = 0.072) chemotherapy treatment (Figure 8C,D). Within the recurrent tumours, *YAP1* mRNA expression was not a significant prognostic factor for patients who did not receive chemotherapy (*p* = 0.62) (Figure 8E); however, low *YAP1* mRNA expression was associated with significantly increased OS in patients who were chemotherapy-treated (*YAP1* high vs. low median survival: 28.05 mo. vs. 16.76 mo., HR (95% CI): 1.742 (1.019–3.168), *p* = 0.043) (Figure 8F). In multivariate analysis, after adjusting for *IDH1* mutation, tumour grade, gender and age, *YAP1* levels were an independent prognostic factor for chemotherapy-treated recurrent astrocytoma (HR (95% CI): 4.06 (1.32–12.27), *p* = 0.013). This indicates the prognostic importance of post-chemotherapy *YAP1* mRNA expression and confirms *YAP1* mRNA expression is not a prognostic factor in primary tumours. It should be noted that the CGGA dataset is comprised of Chinese glioma samples; due to the lack of available recurrent LGG datasets, we cannot extend these findings to the global population.

### 3.10. O6-Methylguanine-DNA Methyltransferase (MGMT) Methylation Status of Astrocytoma Is a Factor in TP53 Mutation/YAP1-Mediated Chemosensitivity

**Figure 9:**
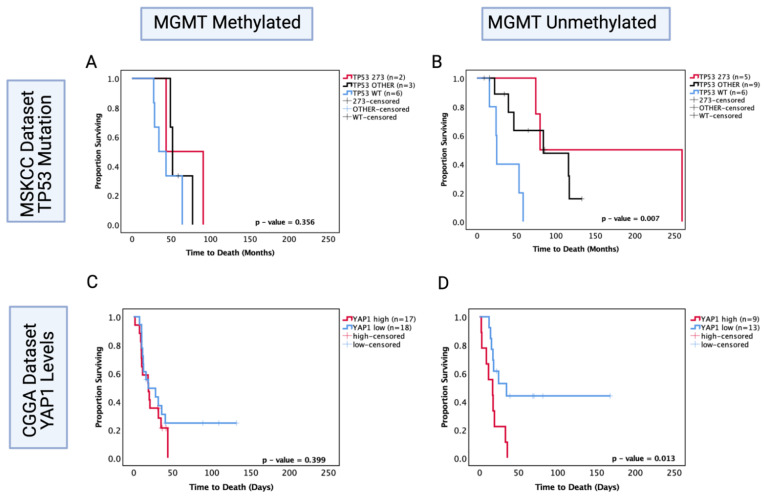
Kaplan–Meier survival curves comparing effects of *TP53* mutation status on the OS of chemotherapy-treated (**A**) *MGMT* methylated and (**B**) *MGMT* unmethylated astrocytoma in the MSKCC dataset. Kaplan–Meier survival curves comparing the effects of YAP1 mRNA expression levels on the OS of chemotherapy-treated (**C**) *MGMT* methylated and (**D**) *MGMT* unmethylated astrocytoma in the CGGA dataset. *TP53* 273: *TP53* codon 273 mutant, *TP53* OTHER: all pathogenic *TP53* mutations except in codon 273, *TP53* WT: wild-type *TP53* status. Log-rank test was used for analysis and a *p*-value < 0.05 for statistical significance.

*MGMT* status is a well-known predictor of chemosensitivity in glioblastoma [47]; thus, in order to understand its role in astrocytoma, we investigated the MSKCC chemotherapy-treated cohort and the CGGA recurrent chemotherapy-treated cohort where the *MGMT* statuses were known and performed survival analysis based on *TP53* mutation and *YAP1* mRNA expression levels, respectively. In the MSKCC chemotherapy-treated cohort, the *MGMT* methylated group showed no significant prognostic effect of *TP53* mutation (*p* > 0.05) (Figure 9A); however, within the *MGMT* unmethylated group, both other *TP53* mutant and codon 273 mutant tumours were associated with a significantly increased OS (other vs. WT median survival: 84.2 mo. vs. 25 mo., HR (95% CI): 0.299 (0.034–0.799), *p* = 0.0.25; codon 273 vs. WT median survival: 170 mo. vs. 25 mo., HR (95% CI): 0.195 (0.039–0.976), *p* = 0.004) (Figure 9B). This pattern of effect was mirrored in the CGGA *YAP1* survival analysis, where *YAP1* mRNA expression was not a significant prognostic marker in the *MGMT* methylated group (*p* = 0.399) (Figure 9C); however, low *YAP1* mRNA was associated with significantly increased OS in chemotherapy-treated *MGMT* unmethylated astrocytoma (*YAP1* high vs. low median survival: 16.76 mo. vs. 30.5 mo., HR (95% CI): 3.078 (1.052–9.01), *p* = 0.013) (Figure 9D).

These data suggest that both codon 273 mutation and *YAP1* mRNA expression may be prognostic markers for the *MGMT* unmethylated group of recurrent astrocytoma treated with chemotherapy. Thus, there may be an association between codon 273 mutant astrocytoma and post-chemotherapy *YAP1* levels. It is, however, important to validate these findings with a larger cohort size where multivariate analysis can also be conducted. Importantly, LGG clinical trial results can be retrospectively re-analysed to determine whether the responders were *MGMT* unmethylated with *TP53* codon 273 mutations and low *YAP1* levels. The findings from this study may, therefore, help to identify the efficacy of chemotherapy treatment based on molecular markers and make LGG therapeutic approaches more targeted.

### 3.11. Possible Mechanism for TP53-Mutation-Dependent Chemosensitivity in Astrocytoma

**Figure 10:**
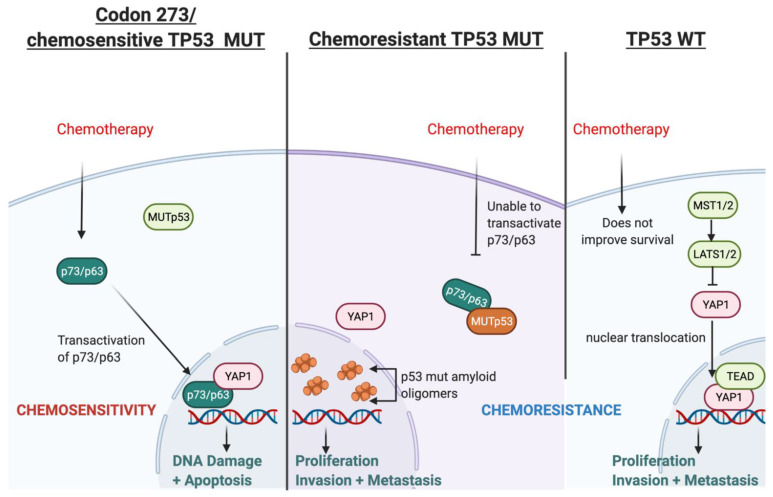
Illustration of the hypothesised mechanism through which *TP53* mutation type determines chemosensitivity in astrocytoma involving transactivated p63/p73 and *YAP1*.

Based on our findings and the current literature, we present a hypothetical mechanism of action of *TP53*-mutation-dependent chemosensitivity in Figure 10, which may involve p73/p63-YAP1-complex-mediated chemosensitivity. Two alternative promoters of TP63 and TP73 lead to different protein variants of p63 and p73: transactivating (TA) isoforms or N isoforms, ΔN-p63 and ΔN-p73, (that lack the N-terminal transactivating domain). The TA proteins, TAp63 or TAp73, retain the tumour-suppressive features activating tumour suppressive genes common to those transactivated by p53. In contrast, the N isoforms can inhibit tumour suppression and act as oncoproteins [48]. Chemotherapy can upregulate the levels of TA proteins and downregulate the N isoforms [49]. The DNA damage response (DDR), induced by chemotherapy, leads to the acetylation and phosphorylation of E2F1, which then upregulates TAp73 expression through the P1 promoter. Various other mechanisms of DDR-dependent TAp73 activation have also been reported [41]. TAp73 then induces apoptosis following genotoxic treatments [50]. On the other hand, ΔN-p63 induces chemo- and radio-resistance in head and neck squamous cell carcinomas via the suppression of p73 [51]; in breast cancer cell lines, the release of TA-p73 is required from ΔN-p63 to enable cisplatin response since ΔN-p63 binds to TA-p73 to inhibit its pro-apoptotic features. Cisplatin dissociates the ΔN-p63/TA-p73 complex via c-Abl-dependent phosphorylation, enabling TAp73 to induce apoptosis [52]. DDR can be induced by temozolomide, a standard chemotherapy for patients diagnosed with astrocytoma (and glioblastoma). This DDR can lead to the transactivation of p63 (TA-p63), thereby suppressing glioblastoma growth and invasion in vitro, which also correlates with a favourable prognosis in GBM [53]. The ratio of ΔN/TA-p73 is a crucial factor in the pro-apoptotic ability of p73 and, ultimately, the efficacy of chemotherapy [54,55].

The type of *TP53* mutation is an important factor in this mechanism because certain *TP53* mutations can inhibit the transactivation of p63 and p73 [56,57,58,59] and affect chemosensitivity [60]; however, *TP53* mutation in codon 273 neither binds to nor inhibits the transcriptional activity of p63/p73 [59,61]. This has been shown in vitro, where codon 273 mutant glioma cells lines were more chemosensitive compared with *TP53* WT cell lines [62,63]. The poorest survival of *TP53* WT astrocytoma in our cohort may relate to this. Furthermore, the accumulation of mutant p53 amyloid oligomers associated with chemoresistance is not present in the codon 273 mutant cell line, whereas increased mutant p53 amyloid oligomers were observed in cells with other *TP53* mutations [64]. Collectively, these reports indicate that certain *TP53* mutations can cause chemoresistance, while *TP53* codon 273 mutation may be associated with chemosensitivity. This may explain the improved clinical outcomes of codon 273 mutant astrocytoma observed in our survival analysis, although it is important to note that these studies were conducted predominantly in glioblastoma and in vitro studies for LGG are warranted to confirm this hypothesis.

*YAP1* may be critically involved in this hypothesised chemosenstivity mechanism because it is a transcriptional co-activator of TAp73 and it may also form complexes with TAp73 to promote apoptosis [41]. These complexes activated apoptosis in a range of cancers following treatment [44,45,46]. *YAP1* has also been shown to stabilise p73 in response to chemotherapy to induce apoptosis [42]. Thus, it is a possible chemosensitivity mechanism in codon 273 mutant astrocytomas.

We have consistently observed decreased survival in *TP53* WT astrocytoma patients, which may be related to chemoresistance in this group. The transactivation of p73/p63 status is unknown in *TP53* WT. However, our analysis showed that gene expression of the Hippo Signalling pathway is upregulated in the *TP53* WT group of TCGA astrocytoma, and PPI analysis showed that the *YAP1* high group (which was also associated with significantly decreased OS) was enriched for *YAP1–TEAD* complexes (Supplementary File 4: Figure S3) that drive transcription and proliferation [65]. This is strongly supported by the GO, DisGenNet and KEGG pathway analyses of this group (Supplementary File 4: Figure S3). Moreover, *YAP1* does not bind with WT p53 [66,67]; hence, it may be available for *YAP1–TEAD* nuclear complex formation. Thus, the *YAP1–TEAD* nuclear complex may counteract the effect of chemotherapy by driving cellular proliferation in *TP53* WT astrocytoma. There is also evidence that p53 and *YAP1* cooperate in chemotherapy efficacy, with direct binding of *YAP1* and p53 to each other’s promoters, increasing their expression in response to chemotherapy in hepatocellular carcinoma cells [68]. *YAP1* can thus affect chemosensitivity through the modulation of p53 [69], which may also depend on p53 mutation status and mutation type. The spectrum of modalities through which *YAP1* interacts, affects and coordinates with p53, p63 and p73 has been reviewed thoroughly [70]. *YAP1* can direct the tumour fate to either proliferation and tumourigenesis (by binding with TEAD in the nucleus and promoting cell survival) [65] or to apoptosis (by interacting/coordinating with p53/p63/p73) [45,71]. Our data supports this, suggesting that the cellular *YAP1* switch may be strongly dependent on p53 mutation status and type. However, the dynamics between *YAP1* and *TP53* post-chemotherapy and this putative mechanism should be validated through well-designed experimental in vitro studies, which is beyond the scope of this paper.

## 4. Discussion

*TP53* is a pivotal gene that is extensively researched across all cancer types due to the high prevalence of *TP53* mutations. In LGG, *TP53* mutations have been reported to be prevalent, particularly in astrocytoma [20,72], which we confirmed in this study. We found that all *TP53* mutations occurred in the region of exons 4 to 8, which is in line with the hotspot region identified in literature [73,74]. The prevalence of *TP53* mutation in astrocytoma (60%) was also similar to previous findings (50–75%) [19,20]. We have found a high prevalence of 18% *TP53* mutation in our oligodendroglioma cohort (9/50), while previous studies have reported up to 34% [19,20]. The significant correlation between *IDH1* mutation and *TP53* mutation is concordant with previous reports [74,75].

Due to the difference in OS between astrocytoma and oligodendroglioma (also confirmed in this study) [76] and the distinct genetic and epigenetic features in these LGG subtypes [77,78,79,80], it is critical to perform separate survival analyses for each histological type. When the original investigations of *TP53* mutation as a prognostic factor in glioma were published between 1991 and 1999 [26,81,82,83,84,85,86,87,88,89], the molecular classifiers of LGG (1p/19q co-deletion, ATRX loss and *IDH1/2* mutations) were not well-established enough to allow LGG sub-typing [90,91,92]. Thus, older studies commonly analysed combined cohorts of either ‘LGG’ or ‘gliomas’ (that also included the much more aggressive glioblastoma) [20,26,73,74,85] and concluded that there was no prognostic effect of *TP53* in LGG or *TP53* mutant cases corresponding to a decreased OS. These results can be attributed to the higher prevalence of *TP53* mutation in astrocytoma, together with the significantly shorter OS of astrocytoma, compared to oligodendroglioma.

In our study of 102 LGG specimens, separate survival analysis was performed for astrocytoma and oligodendroglioma, and only confirmed ‘pathogenic’ mutations were considered for the analyses. Hotspot mutations were accounted for in the survival analysis, which will be discussed later in this discussion. The results comparing combined *TP53* mutant vs. *TP53* WT patients showed significantly increased OS for *TP53* mutant astrocytoma patients. Survival analysis of the TCGA astrocytoma dataset confirmed our findings. In LGG, this is the first report of *TP53* mutation being a favourable prognostic factor, although this was reported previously in other cancers, including glioblastoma [93,94], sarcomas [95], ovarian serous cystadenocarcinoma [96] and cervical cancer [97]. In multivariate analysis adjusting for *IDH1* mutation, age, gender and tumour grade, the combined *TP53* mutation showed a trend for improved survival compared to WT, leading us to believe there are specific *TP53* mutations accounting for the improved survival in the univariate analysis. We thus investigated specific hotspot *TP53* mutations.

Hotspot mutations in *TP53* codon 273 were found in 33% of astrocytoma tumours in our cohort. These codon 273 mutations have been reported in LGG cohorts previously [84,98,99,100,101]. *TP53* codon 273 mutations are hotspot mutations reported frequently in other cancers. R273H and R273C are contact mutations in the DNA-binding domain that disrupt DNA contact points by changing the structure of the protein and ultimately prevent DNA binding [102]. In the current study, we showed that in the cohort of astrocytoma, *TP53* codon 273 mutations were significantly associated with prolonged OS compared to WT in both univariate and multivariate analysis. It is possible that codon 273 mutation (along with other *TP53* mutations that may have behaved similarly, such as the codon 273 mutations) has contributed to the trend for the improved survival effect of the combined pathogenic *TP53* mutations observed in our analysis.

A number of more recent studies have performed separate analyses for the histological sub-types and found no significant prognostic effect of *TP53* status [20,103]. These studies used smaller or equal sized cohorts to our current study, and they did not incorporate *TP53* mutation ‘pathogenic status’ in their survival analysis. Moreover, according to our findings, the frequency of *TP53* codon 273 mutations in their cohorts may have affected their survival results. Interestingly, the most recent prognostic investigation of *TP53* in adult LGG (cohort size, n = 61) [19] did not have any tumour harbouring a mutation in codon 273, which we found to be a hotspot mutation in our cohort. Moreover, due to the smaller number of astrocytoma in this published cohort (n = 8), the cohort was analysed as a combined cohort of ‘LGG’, and the study showed no significant prognostic effect of *TP53* mutation. These examples suggest that cohort size, distribution of histological types, inclusion of ‘pathogenic’ mutations, and frequency of specific hotspot mutations in the cohorts play a crucial role in determining the prognostic value of the *TP53* mutations.

To our knowledge, only one group, Peraud et al., has performed PFS analysis on LGG based on different *TP53* codons, where they reported that PFS depended on the specific codon of *TP53* mutation. Interestingly their study showed that codon 273 mutants had significantly increased PFS compared to codon 175 mutants [101]. However, *TP53*-WT cases were not included in the PFS analysis, and no codon-based OS analysis was performed. Additionally, their cohort included ‘oligoastrocytoma’, which is now considered a misdiagnosis of either oligodendroglioma or astrocytoma.

The MSKCC and TCGA datasets were analysed to confirm our findings, both of which showed that *TP53* mutations (codon 273 and other) were significantly associated with increased OS and PFS. This is the first report of a specific *TP53* codon mutation carrying OS and PFS prognostic value in adult LGG. Interestingly, a study of GBM (n = 108) reported significantly favourable prognosis for *TP53* mutant GBM patients (n = 28). Further analysis of these data showed that the most frequent *TP53* mutations in their cohort were in codon 273 [93].

In oligodendroglioma, we found *TP53* mutation was associated with a significantly decreased OS in the current cohort using univariate and multivariate analyses. The TCGA dataset did not corroborate with this; however, specifically, codon 273 mutation was associated with a significantly decreased OS in both univariate and multivariate analyses of the TCGA oligodendroglioma cohort. The opposite prognostic effect of *TP53* mutation in oligodendroglioma compared to astrocytoma represents the critical molecular and mechanistic differences between the two histological sub-types and emphasises the need to analyse these sub-types as separate cohorts in transcriptomic and survival studies.

Our investigation showed that codon 273 mutant astrocytomas may be more chemosensitive compared to *TP53* WT as the OS of codon 273 mutant astrocytoma was significant longer in the chemotherapy-treated group, but not in the ‘no-chemotherapy’ group in two independent cohorts. *YAP1*, which has been previously shown to play a role in inducing sensitivity to treatment [44,45,46], correlated with *TP53* expression in the chemotherapy-treated group of LGG only, suggesting the possibility of modulated interactions between p53 and *YAP1* post-chemotherapy. Supporting these findings, we found that *YAP1* expression was an independent prognostic factor in the chemotherapy-treated group of astrocytoma only. In a study of 117 glioma tissues, high *YAP1* protein expression was previously correlated with more aggressive glioma phenotypes and survival. However, it is unclear whether these tissues were from primary or recurrent tumours [104]. It is important to analyse recurrent tumours post-chemotherapy to identify fate-driving transcriptomic changes post-treatment. Our findings suggest a role for *YAP1* (and the interactions of *YAP1* and *TP53*) in chemotherapy efficacy, and we presented a hypothesised chemosensitivity mechanism involving *TP53*-mutation-type-dependent modulation of TAp63/p73 and *YAP1*, leading to divergent chemotherapeutic outcomes, which will be explored and validated by our team in future studies. DEG, GO, KEGG pathway and DisGenNet analysis of recurrent chemotherapy-treated astrocytoma also supported our proposed mechanism of chemosensitivity. A limitation in this study, however, is the inability to understand the effect of radiotherapy since it was administered both with/without chemotherapy. However, our data showed consistent results when stratified by chemotherapy status only, suggesting that chemotherapy efficacy is more dominantly associated with the molecular markers investigated compared to radiotherapy.

We further observed the potential role of *MGMT* methylation in this study as *YAP1* mRNA expression and codon 273 mutation were particularly prognostic in the *MGMT* unmethylated sub-group of astrocytoma patients treated with chemotherapy. Thus, we identified a *TP53*-mutation-dependent and *YAP1*-level-dependent chemosensitive sub-group within *MGMT* unmethylated astrocytoma. This is interesting because *MGMT* methylation has been significantly associated with certain types of *TP53* mutation in astrocytoma [105], yet again hinting a possible link between *TP53* mutation type and *YAP1*-mediated chemosensitivity. Validation with a larger cohort or retrospective analysis of past clinical trials may provide more definitive data to aid effective chemotherapeutic treatment decisions in astrocytoma.

In summary, this study reports for the first time the improved prognostic significance of *TP53* mutation status in adult LGG and also the importance of considering the effect of specific *TP53* mutation types/codon-wise mutations. The hotspot *TP53* codon 273 mutations were associated with prolonged survival in astrocytoma patients compared with *TP53* WT and other *TP53* mutations.

## 5. Conclusions

For many years, the prognostic role of *TP53* mutation in LGG has been unclear. Recent knowledge of molecular features, access to mutation databases and considerably large cohorts have enabled this study to uncover the significant prognostic role of *TP53* in astrocytoma. We conclude that *TP53* plays an important role in astrocytoma biology and treatment response, and *TP53* mutation types can have diverse effects in gliomagenesis post-treatment. Furthermore, we identified sub-groups (codon 273 mutant and/or low *YAP1* level) of *MGMT* unmethylated recurrent chemotherapy-treated astrocytoma that responded well to chemotherapy. Our data also suggest that *TP53* WT astrocytoma tumours, which did not respond well to chemotherapy, need new targeted treatments to improve the unfavourable OS. Developing better treatments for recurrent tumours is vital in prolonging the OS of astrocytoma patients, and obtaining and analysing transcriptomic data of recurrent tumours will be a practical approach to aid in this process.

Retrospective genetic analysis of drug responders and non-responders in clinical trials can elucidate the chemosensitivity score of codon 273 mutant astrocytoma patients. There is a lack of specialised and targeted treatment options for recurrent astrocytoma; hence, the development of therapeutic options for this group is key to prolonging survival. Our data suggest that *YAP1* should be further investigated as a potential druggable target in the sub-group of recurrent chemotherapy-treated *MGMT* unmethylated astrocytoma.

## Figures and Tables

**Table 1:** Patient and tumour characteristics.

Age at diagnosis, median ± SD (years)	36 ± 10	
Median follow-up (years, 95% CI)	9.0 (7.9–10.1)	
Gender, n (%)	F	53 (51.9%)
	M	49 (48.0%)
Diagnosis, n (%)	Astrocytoma grade II	32 (31.1%)
	Astrocytoma grade III	19 (18.4%)
	Oligodendroglioma grade II	26 (26.2%)
	Oligodendroglioma grade III	25 (24.3%)
*IDH1* mutation, n (%)	Wild-type	17 (17%)
	Mutant	85 (83%)
Anatomical Location of Tumour, n (%)	Frontal	45 (44.1%)
	Insular	6 (5.9%)
	Motor Cortex	1 (1.0%)
	Parietal	13 (12.7%)
	Temporal	22 (21.6%)
	Unknown	15 (14.7%)
Treatment, n (%)	Surgery and chemoradiation	28 (27.5%)
	Surgery and chemotherapy	15 (14.7%)
	Surgery and radiotherapy	11 (10.8%)
	Surgery only	27 (26.5%)
	Unknown adjuvant therapy	21 (20.5%)

**Table 2:** *TP53* mutation information and their pathogenic status.

Mutation Syntax	Amino Acid Change	Frequency	Type of Mutation	Ref SNP ID	Outcome
c.215C>G	p.P72R	24	Missense	rs1042522	Neutral
c.321C>G	p.Y107 *	1	Nonsense	rs770776262	Neutral
c.377A>G	p.Y126C	2	Missense	rs1555526335	Pathogenic
c.469_470delGT		1	Frameshift Deletion	Novel mutation	Pathogenic
c.473G>A	p.R158H	1	Missense	rs587782144	Pathogenic
c.476C>A	p.A159D	1	Missense	unavailable	Pathogenic
c.480G>A	p.M160I	1	Missense	rs193920817	Pathogenic
c.488A>G	p.Y163C	1	Missense	rs148924904	Pathogenic
c.517G>C	p.V173L	1	Missense	rs876660754	Pathogenic
c.524G>C	p.R175P	2	Missense	rs28934578	Pathogenic
c.526T>G	p.C176G	1	Missense	rs967461896	Pathogenic
c.537T>A	p.H179Q	1	Missense	rs876660821	Neutral
c.581T>G	p.L194R	1	Missense	rs1057519998	Pathogenic
c.639A>G	p.R213R	3	Silent	rs1800372	Neutral
c.643A>G	p.S215G	1	Missense	rs886039484	Pathogenic
c.659A>G	p.Y220C	2	Missense	rs121912666	Pathogenic
c.700T>C	p.Y234H	1	Missense	rs864622237	Pathogenic
c.711G>A	p.M237I	1	Missense	rs587782664	Pathogenic
c.734G>A	p.G245D	1	Missense	rs121912656	Pathogenic
c.742C>T	p.R248W	1	Missense	rs121912651	Pathogenic
c.743G>A	p.R248Q	1	Missense	rs11540652	Pathogenic
c.797G>A	p.G266E	1	Missense	rs193920774	Pathogenic
c.817C>T	p.R273C	15	Missense	rs121913343	Pathogenic
c.818G>A	p.R273H	3	Missense	rs28934576	Pathogenic
c.823T>G	p.C275G	1	Missense	rs1029688274	Pathogenic
c.861G>A	p.E287E	1	Silent	rs748891343	Neutral

* Substitution—nonsense mutation.

**Table 3:** Clinicopathological features and *TP53* mutation status.

		*TP53* Mutation Status		*p*-Value
		Wild-Type	Mutant	
		n (%)	n (%)	
Age at diagnosis, mean ± SD ^1^, (years)		38 ± 11	34 ± 9	**0.046**
Gender	F	32 (52%)	20 (50%)	0.694
	M	29 (48%)	20 (50%)	
Diagnosis	Astrocytoma II	8 (13%)	24 (60%)	<**0.001**
	Astrocytoma III	12 (19%)	7 (18%)	
	Oligodendroglioma II	21 (34%)	5 (12%)	
	Oligodendroglioma III	20 (34%)	4 (10%)	
*IDH1* mutation status	Wild-type	14 (23%)	2 (5%)	**0.009**
	Mutant	47 (77%)	37 (95%)	
1p/19q co-deletion status	Co-deleted	22 (36%)	0 (0%)	<**0.001**
	Non-co-deleted	9 (15%)	18 (45%)	
	Unknown	30 (49%)	22 (55%)	
*p53* expression level ^2^	Unknown	22 (36%)	17 (43%)	<**0.001**
	Low	25 (41%)	1 (1%)	
	High	14 (23%)	22 (55%)	
Treatment	Surgery and chemoradiation	16 (26%)	12 (30%)	**0.016**
	Surgery and chemotherapy	10 (16%)	5 (13%)	
	Surgery and radiotherapy	8 (13%)	3 (7%)	
	Surgery only Unknown	21 (35%)	6 (15%)	
		6 (10%)	14 (35%)	

^1^ SD = standard deviation; ^2^ p53 expression considered high >10%.

**Table 4:** Univariate and multivariate survival analyses of astrocytoma in this study.

	Overall Survival			
	Univariate Analysis		Multivariate Analysis	
*TP53* Mutation	Hazard Ratio (95% CI)	*p*-Value	Hazard Ratio (95% CI)	*p*-Value
*TP53* combined vs. WT	0.331 (0.138–0.796)	**0.014**	0.392 (0.132–1.163)	0.091
*TP53* codon 273 vs. other	0.466 (0.172–1.262)	0.133	0.175 (0.04–0.759)	**0.02**
*TP53* codon 273 vs. WT	0.232 (0.076–0.711)	**0.011**	0.169 (0.036–0.766)	**0.021**
Other vs. WT	0.500 (0.150–1.590)	0.241	1.042 (0.278–3.906)	0.951

**Table 5:** Univariate survival analysis of TCGA and MSKCC datasets.

Dataset	*TP53* Mutation	Hazard Ratio (95% CI)	*p*-Value
TCGA OS	*TP53* combined vs. WT	0.199 (0.113–0.350)	**<0.0001**
	Codon 273 vs. WT	0.182 (0.0069–0.479)	**<0.001**
	Other vs. WT	0.210 (0.116–0.379)	**<0.0001**
TCGA PFS	*TP53* combined vs. WT	0.336 (0.218–0.517)	**<0.0002**
	Codon 273 vs. WT	0.260 (0.125–0.539)	**<0.0001**
	Other vs. WT	0.360 (0.229–0.567)	**<0.0001**
MSKCC OS	Codon 273 vs. WT	0.124 (0.053–0.289)	**<0.0001**
	Other vs. WT	0.231 (0.136–0.392)	**<0.0001**
MSKCC PFS	Codon 273 vs. WT	0.154 (0.83–0.286)	**<0.0001**
	Other vs. WT	0.187 (0.117–0.297)	**<0.0001**

## Data Availability

The datasets generated and analysed in this study are available from the corresponding author.

## Supplementary Materials

The following are available online at https://www.mdpi.com/article/10.3390/cancers13215362/s1, Supplementary File 1 in Table S1: PCR conditions and primer sequences for *TP53* sequencing. Supplementary File 2 in Figure S1: Kaplan–Meier survival curves stratified by (A) *IDH1* mutation status, (B) type of LGG, and (C) *TP53* mutation status for the combined cohort of astrocytoma and oligodendroglioma. Supplementary File 3 in Figure S2: Volcano plot portraying differentially expressed genes between *YAP1* high and *YAP1* low groups in CGGA recurrent chemotherapy-treated astrocytoma. Supplementary File 4 in Figure S3: GO enrichment terms of differentially expressed genes, KEGG pathway, and DisGenNet analysis of *YAP1* low (A,C,E) and *YAP1* high (B,D,F), respectively. Top three PPI network modules of *YAP1* low (G) and *YAP1* high groups (H).
