# Supplementary material for: TP53 Mutation Is a Prognostic Factor in Lower Grade Glioma and May Influence Chemotherapy Efficacy
Source: Cancers (Basel). 2021 Oct 26;13(21):5362. doi: 10.3390/cancers13215362 (PMC8582451; doi:10.3390/cancers13215362)
Supplement: Supplementary file 1 [file cancers-13-05362-s001.zip › Supplementary File 1.pdf]

# Supplementary File 1

Table S1: PCR conditions and primer sequences for *TP53* sequencing

| Exon | Annealing Temperature ◦C | Primer Sequences 5’ to 3’ |
|---|---|---|
| 1 and 2 | 64 | *Forward* – CTCATGCTGGATCCCCACTTTTC<br>*Reverse* - CCCAGCCCTCCAGGTCCCCAGCCC |
| 3 | 66.4 | *Forward* - GAGGGCTGGGGGGCTGGGGGGCTG<br>*Reverse* - GGGGGATACGGCCAGGCATTGAAG |
| 4 | 66.4 | *Forward* – GCCGTCTTCCAGTTGCTTTATCTG<br>*Reverse* - GCAATCAGTGAGGAATCAGAGGCC |
| 5 | 68 | *Forward* - GCAGCTGGGGCTGGAGAGACGAC<br>*Reverse* - CTCACCTGGAGGGCCACTGACAAC |
| 6 | 60.4 | *Forward* - CCTGCTTGCCACAGGTCT<br>*Reverse* - GTGTGCAGGGTGGCAAGT |
| 7 | 56.7 | *Forward* - CAAGGGTGGTTGGGAGTAGATGG<br>*Reverse* - GAAAGGTGATAAAAGTGAATCTG |
| 8 | 64 | *Forward* - GCAAGCAGGACAAGAAGCGGTGG<br>*Reverse* - CCATTGTCTTTGAGGCATCACTGC |
| 9 | 50.5 | *Forward* - CTAAATGCATGTTGCTTTTGTAC<br>*Reverse* - GAATGGAATCCTATGGCTTTCC |
| 10 | 64 | Forward - GCCCTTCAAAGCATTGGTCAGGG<br>*Reverse* - GCAAGGGTTCAAAGACCCAAAACC |
