# Supplementary material for: TP53 Mutation Is a Prognostic Factor in Lower Grade Glioma and May Influence Chemotherapy Efficacy
Source: Cancers (Basel). 2021 Oct 26;13(21):5362. doi: 10.3390/cancers13215362 (PMC8582451; doi:10.3390/cancers13215362)
Supplement: Supplementary file 1 [file cancers-13-05362-s001.zip › Supplementary File 2.pdf]

Supplementary File 2

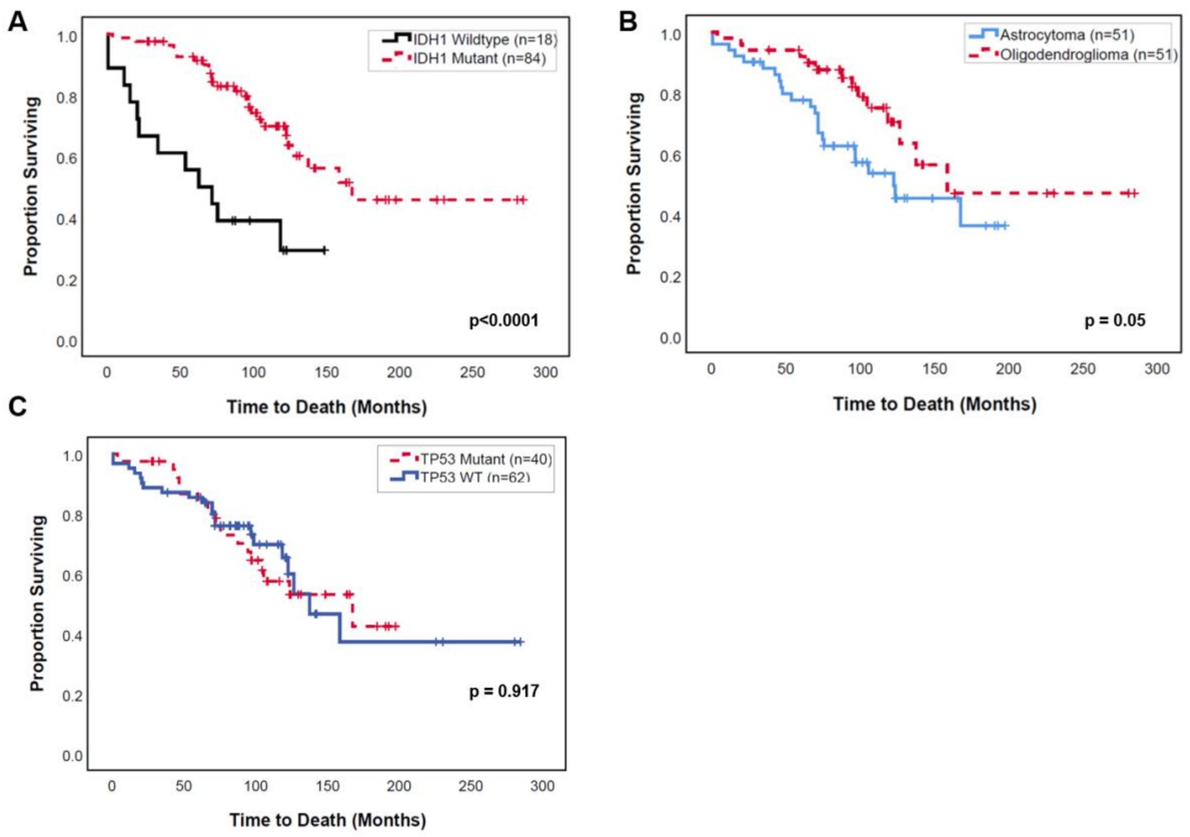

Figure S1. Kaplan-Meier survival curves stratified by (A) *IDH1* mutation status (B) type of LGG (C) *TP53* mutation status for the combined cohort of astrocytoma and oligodendroglioma. WT: Wildtype.
