# Supplementary material for: TP53 Mutation Is a Prognostic Factor in Lower Grade Glioma and May Influence Chemotherapy Efficacy
Source: Cancers (Basel). 2021 Oct 26;13(21):5362. doi: 10.3390/cancers13215362 (PMC8582451; doi:10.3390/cancers13215362)
Supplement: Supplementary file 1 [file cancers-13-05362-s001.zip › Supplementary File 3.pdf]

Supplementary File 3

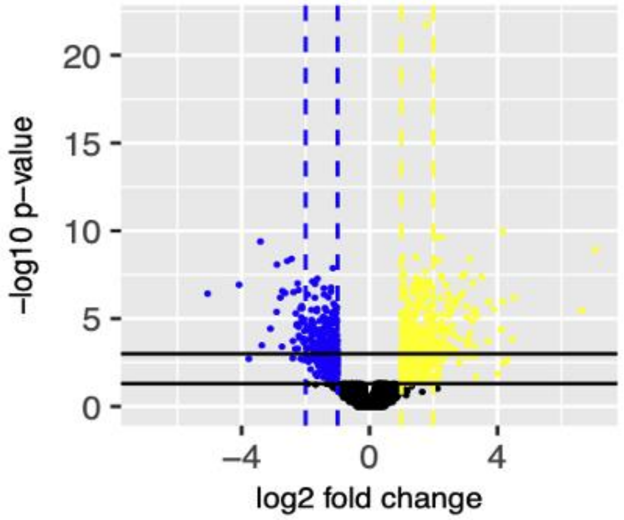

Figure S2: Volcano plot portraying differentially expressed genes between *YAP1* high and *YAP1* low groups in CGGA recurrent chemotherapy treated astrocytoma.
