# Supplementary material for: TP53 Mutation Is a Prognostic Factor in Lower Grade Glioma and May Influence Chemotherapy Efficacy
Source: Cancers (Basel). 2021 Oct 26;13(21):5362. doi: 10.3390/cancers13215362 (PMC8582451; doi:10.3390/cancers13215362)
Supplement: Supplementary file 1 [file cancers-13-05362-s001.zip › Supplementary File 4.pdf]

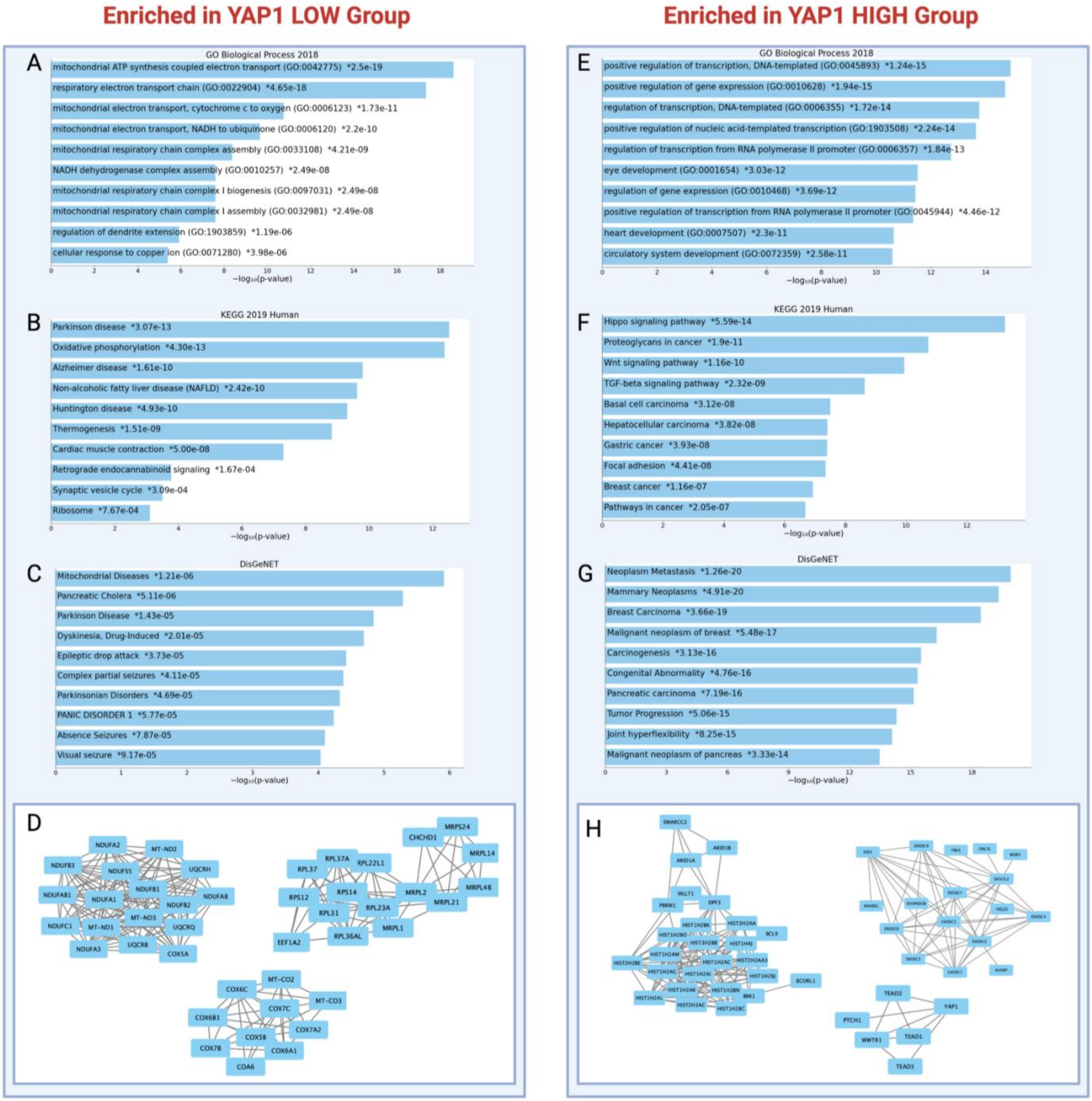

Figure S3 GO enrichment terms of differentially expressed genes, KEGG pathway, DisGenNet analysis of *YAP1* low **(A, C, E)** and *YAP1* high **(B, D, F)** respectively. Top three PPI network module of *YAP1* low **(G)** and *YAP1* high groups **(H)**.
